# Comparison of midwife-led and consultant-led care of healthy women at low risk of childbirth complications in the Republic of Ireland: a randomised trial

**DOI:** 10.1186/1471-2393-11-85

**Published:** 2011-10-29

**Authors:** Cecily Begley, Declan Devane, Mike Clarke, Colette McCann, Patricia Hughes, Mary Reilly, Roisin Maguire, Shane Higgins, Alan Finan, Siobhan Gormally, Miriam Doyle

**Affiliations:** 1 School of Nursing and Midwifery, Trinity College Dublin, Dublin 2, Ireland; 2 School of Nursing and Midwifery, National University of Ireland, Galway, Ireland; 3 All-Ireland Hub for Trials Methodology Research, Queen's University Belfast, Northern Ireland; 4 Our Lady of Lourdes Hospital, Drogheda, Ireland; 5 Coombe Women and Infant's University Hospital, Dublin 2, Ireland; 6 Cavan General Hospital, Cavan, Ireland; 7 Louth County Hospital, Dublin Road, Dundalk, Co. Louth, Ireland; 8 National Maternity Hospital, Dublin 2, Ireland; 9 Midland Regional Hospital, Portlaoise, Ireland

## Abstract

**Background:**

No midwifery-led units existed in Ireland before 2004. The aim of this study was to compare midwife-led (MLU) versus consultant-led (CLU) care for healthy, pregnant women without risk factors for labour and delivery.

**Methods:**

An unblinded, pragmatic randomised trial was designed, funded by the Health Service Executive (Dublin North-East). Following ethical approval, all women booking prior to 24 weeks of pregnancy at two maternity hospitals with 1,300-3,200 births annually in Ireland were assessed for trial eligibility.1,653 consenting women were centrally randomised on a 2:1 ratio to MLU or CLU care, (1101:552). 'Intention-to-treat' analysis was used to compare 9 key neonatal and maternal outcomes.

**Results:**

No statistically significant difference was found between MLU and CLU in the seven key outcomes: caesarean birth (163 [14.8%] *vs *84 [15.2%]; relative risk (RR) 0.97 [95% CI 0.76 to 1.24]), induction (248 [22.5%] *vs *138 [25.0%]; RR 0.90 [0.75 to 1.08]), episiotomy (126 [11.4%] *vs *68 [12.3%]; RR 0.93 [0.70 to 1.23]), instrumental birth (139 [12.6%] *vs *79 [14.3%]; RR 0.88 [0.68 to 1.14]), Apgar scores < 8 (10 [0.9%] *vs *9 [1.6%]; RR 0.56 [0.23 to 1.36]), postpartum haemorrhage (144 [13.1%] *vs *75 [13.6%]; RR 0.96 [0.74 to 1.25]); breastfeeding initiation (616 [55.9%] *vs *317 [57.4%]; RR 0.97 [0.89 to 1.06]). MLU women were significantly less likely to have continuous electronic fetal monitoring (397 [36.1%] *vs *313 [56.7%]; RR 0.64 [0.57 to 0.71]), or augmentation of labour (436 [39.6%] *vs *314 [56.9%]; RR 0.50 [0.40 to 0.61]).

**Conclusions:**

Midwife-led care, as practised in this study, is as safe as consultant-led care and is associated with less intervention during labour and delivery.

**Trial registration number:**

ISRCTN: ISRCTN14973283

## Background

Maternity care in Ireland is predominantly hospital-based and consultant-led. In the early 21st century, it is provided free of charge to all women attending the 'public', hospital-based, service. Approximately 50% of women have private insurance that covers, or partially covers, the costs for them to attend for care on a 'semi-private' or 'private' basis. The three types of care differ as follows:

• Public: cared for antenatally by a team of doctors led by a consultant, cared for in labour and at birth by a team of qualified and student midwives, under the supervision of obstetricians, cared for in the postnatal period in hospital in a public ward, usually with 5 or more beds, for 2-3 days.

• Semi-private: cared for antenatally by a non-consultant qualified obstetrician, cared for in labour and at birth by a team of qualified and student midwives, under the supervision of the obstetrician on call, cared for in the postnatal period in hospital in a semi-private, usually 2-4-bedded ward, for 2-3 days. Not available in all hospitals.

• Private: cared for antenatally by a chosen consultant obstetrician, cared for in labour and at birth by a team of qualified and student midwives, under the supervision of the obstetrician or their designated replacement, who will often be present for the birth, cared for in the postnatal period in hospital in a private room for 2-5 days.

Some hospitals provide 'out-reach' antenatal and postnatal care clinics, but there are no national community midwifery services available in the public health system, providing either antenatal or postnatal care. The Public Health Nursing service provides a minimum of one home visit, usually within 2 days of discharge from hospital, and further visits if necessary. Women attend their general practitioner (GP) at six weeks for a check-up of themselves and their baby. A small number of self-employed community midwives care for women having a home birth, (n = 148 births, 0.2% of the total 76,021 births in 2009) [1].

This hospital-based, consultant-led model of maternity care in Ireland has been described as 'highly medicalised' [2] typified by the use of various forms of 'active management of labour' (AML) in most of the 20 maternity units in Ireland. AML includes involvement of a consultant obstetrician, one-to-one midwifery care and use of routine artificial rupture of membranes (ARM) and intravenous oxytocin if a woman's cervix is not dilating by one centimetre per hour [3]. It has been suggested that the main beneficial effect of AML may arise from continuous support of a midwife rather than routine use of amniotomy and oxytocin [3]. Certainly, early routine ARM, one facet of AML, has been criticised as "not scientifically justified" [4] and a Cochrane Review found "no shortening of the length of first stage of labour and a possible increase in caesarean section", concluding "routine amniotomy is not recommended for normally progressing labours or in labours which have become prolonged" [5]. Another Cochrane Review found that AML was associated with a small decrease in caesarean section (CS), but that it was "highly prescriptive and interventional" [6] and the authors advised further research to determine the acceptability of AML to women [6].

Other models, including midwife-led care, tend to be less prescriptive and are founded on the principle of childbirth being a normal, physiological yet life-changing event. 'Midwife-led care' is defined as care where midwives are, "in partnership with the woman, the lead professional with responsibility for assessment of her needs, planning her care, referral to other professionals as appropriate, and for ensuring provision of maternity services" [7]. Midwife-led units (MLUs) are seen as an important and necessary development in care [8] and offer the majority of women a woman-centred, [9,10] cost-effective, [10] safe [11] and satisfying [9,10,12,13] alternative to consultant-led care. The most recent published version of the Cochrane Review comparing midwife-led with other forms of care in pregnancy and childbirth involved 11 trials including 12,276 women. Six studies included women at low risk of complications and five recruited women of high, or mixed, levels of risk. Women who received midwife-led care were less likely to have fetal/neonatal loss before 24 weeks, episiotomy, regional analgesia/anaesthesia, instrumental birth, or antenatal hospitalisation; and were more likely to have no intrapartum analgesia/anaesthesia, spontaneous vaginal birth, to feel in control, to be attended at birth by a known midwife and to initiate breastfeeding. Infants of mothers randomised to midwife-led care had a shorter mean length of neonatal hospital stay. No difference was found in antepartum haemorrhage, mean number of antenatal visits, overall fetal loss and neonatal death, fetal loss or neonatal death ≥ 24 weeks, amniotomy, labour augmentation, mean length of labour, induction of labour, use of opiate analgesia, caesarean section, perineal laceration requiring suturing, intact perineum, postpartum haemorrhage, duration of postnatal hospital stay, low birth weight infant, preterm birth, Apgar scores ≤ 7 at 5 minutes, admission of infant to special care or neonatal intensive care, neonatal convulsions, or maternal postpartum depressions. The review concludes "most women should be offered midwife-led models of care and women should be encouraged to ask for this option" [7]. In Ireland, there were no MLUs before 2004, and few examples of midwife-led care. In 2001, the Kinder Report on women's health services in the North-Eastern region emphasised the need for evidence-based care and recommended that MLUs be established in Cavan and Drogheda [14]. Amid uncertainty as to how far the findings of the Cochrane Review on midwife-led care could be applied to an Irish population, the former North-Eastern Health Board (NEHB) (now the Health Service Executive, Dublin North-East (HSE-DNE)) planned the introduction of 'alongside' midwife-led units within a randomised trial (the "MidU" study). The aim was to compare the effects of midwife-led care (in an MLU) with consultant-led care for healthy women without risk factors for labour and delivery, on rate of interventions, maternal satisfaction, and neonatal and maternal outcomes. In addition, the costs of both types of care were to be measured and compared. Rate of interventions, neonatal and maternal outcomes are presented here; cost-effectiveness and maternal satisfaction will be presented in future papers.

## Methods

### Study setting and participants

Two MLUs were constructed in Our Lady of Lourdes Hospital (OLOL) in Drogheda (3,200 births per year) and Cavan General Hospital (CGH), Cavan (1,300 births per year), both located in large towns (28,000 and 4,000 inhabitants respectively) serving a semi-urban and rural population, of mixed race, with white Irish in the majority. Both units were housed within their parent hospital in re-furbished existing accommodation, close to the main labour ward, and aimed to provide an integrated service using evidence-based guidelines and procedural policies. Twelve staff midwives were employed in OLOL MLU and seven in CGH.

This two group, two-centre, pragmatic randomised trial was conducted between July 2004 and June 2007, with a pilot study for the first seven months, which refined the eligibility criteria and practice guidelines. No changes were made to methods after trial commencement. Recruitment to the main study took place from February 2005 to November 2006, with the last birth in June 2007 when the full sample size had been reached. The null hypothesis stated that "there will be no difference found between midwife-led care and consultant-led care for healthy women without risk factors for labour and delivery as measured by rate of interventions, maternal satisfaction and neonatal and maternal morbidity outcomes".

Information on the MLU service and study invitations were sent by post, or via their GP, to women availing of public care in the HSE-DNE. At the booking clinic, women who had not completed 24 weeks of pregnancy were assessed for trial eligibility by midwives, using multi-disciplinary agreed guidelines (Table 1), and had all questions answered. Those who agreed to participate gave written consent.

**Table 1:** Trial eligibility - maternal exclusion criteria

• ≥ 40 years of age and ≤16 years age at delivery • Grand multiparity (> 5) • Height: < 152 cms (5 feet) • BMI < 18 or > 29 • Medical History: respiratory, renal, infective, immune, neurological, cardiovascular, gastrointestinal, endocrine, haematological, mental ill-health, muscoskeletal • Social Current history of drug misuse Smoking ≥ 20 cigarettes per day • Latex allergy	• Previous obstetric history History of preterm birth at < 34 weeks gestation, recurrent miscarriage, moderate to severe pre-eclampsia, intra-uterine growth restriction, previous stillbirth, CS, eclampsia, uterine rupture, placental abruption, PUPP, obstetric cholestasis, 3rd or 4th degree tear, PPH (> 500 mls or symptomatic), manual removal of placenta, shoulder dystocia, midtrimester miscarriage, neonatal death, infant with hypoxic ischaemic encephalopathy	• Previous gynaecological history Uterine surgery, myomectomy, hysterotomy, cone biopsy (unless subsequent term vaginal delivery), two previous Letz procedures, uterine fibroids, cervical cerclage, infertitlity, uterine anomaly, perineal reconstruction (more than 24 hours post birth)

The Faculty of Health Sciences Research Ethics Committee of Trinity College Dublin and the former NEHB approved the study. An independent Data and Safety Monitoring Board (DSMB) was established and conducted an interim analysis on data on the first 495 women in the main study (33%), with a stopping guideline alpha of 0.001 [15]. The DSMB found insufficient evidence of benefit or harm in either group and recommended the study should continue. The second interim analysis did not take place as the full sample size had already been recruited by the time manual collection of all data on the first 66% of the planned sample size had been completed.

### Randomisation and masking

Women were randomised to a MLU or consultant-led unit (CLU) in a two to one ratio (to make cost-effective use of the refurbished MLUs and allocated staff), using an independent telephone randomisation service. Random sequences of block sizes of two, three, four or five were used, stratified by study centre using a separate block randomisation list for each of the two centres, and by random permutations of group allocation within each block. Block sizes were concealed until completion of the trial. Random integers were obtained using a random number generator available in StatsDirect [16]. The enrolling midwife logged demographics, eligibility, consent and contact details, provided this to the randomisation service and was then informed of the allocation (MLU or CLU) and the unique study number.

As there was no access to MLU care except through the study, carers were aware that all women in the MLU were included in MidU. Therefore, identification of women randomised to the CLU group was not masked, as blinding participants allocated to control groups when it is impossible to blind those in experimental groups has been criticised [17]. Ensuring that all women, and their carers, were aware of their trial status should minimise the impact of this on differences in outcomes between the groups.

### Procedures

Women randomised to CLU received standard care: antenatal care provided by obstetricians and, if desired, by the woman's GP, supported by the hospital medical team with assistance from midwives, who did not usually perform assessment; intrapartum care provided by midwives unless complications developed, with consultant overview; and postpartum care (2-3 days in hospital) provided by midwives, overseen by consultants. Women were discharged into the care of Public Health Nurses.

Women randomised to MLU received midwife-led care where care was provided by the same small group of midwives throughout pregnancy, birth and into the postnatal period. Antenatal care (including assessment) was provided by midwives in the unit, or in an out-reach clinic and, if desired, by the woman's GP. Where complications arose, women were transferred to CLU based on agreed criteria (Table 2). Following obstetric assessment women transferred back to MLU or remained in CLU as appropriate, where they received the usual care described above. Intrapartum care was provided by midwives in the MLU with transfer to CLU if necessary, based on agreed criteria (Table 3). Postnatal care was by midwives in the MLU for up to two days, with transfer of women or neonates to CLU if necessary (and back, as appropriate), based on agreed criteria (Table 4). On discharge, MLU midwives visited at home, and/or provided telephone support, up to the seventh postpartum day, when care was transferred to the Public Health Nursing service. Care in the MLUs was provided by the full team of midwives (12 in OLOL and 7 in CGH), so women did not necessarily have the degree of continuity of care that might be expected from case-load models of midwife-led care.

**Table 2:** Transfer criteria during pregnancy

Maternal	Maternal	Maternal	Fetal
• Rhesus disease	• Prolonged pregnancy i.e. > 40+10	• Gestational diabetes	• Clinically suspected small for gestational age baby
• Atypical antibodies	• Pre-term spontaneous rupture of the membranes	• Pre-labour rupture of membranes at term for > 48 hrs	• Known fetal anomaly
• Antepartum haemorrhage	• Gestational Hypertension (≥ 140/90 mmHg)	• Induction of labour	• Oligo-hydramnious
• Multiple pregnancy	• Eclampsia	• Symptomatic vaginal discharge.	• Poly-hydramnious
• Maternal request for prenatal screening	• Pre-eclampsia	• Unbooked pregnancy	• Reduced fetal movements
• Plancental abruption	• Proteinuria ≥ 1+ on repeat specimen at same visit.	• Group B Strep	
• Unstable lie	• Suspected thromboembolism	• More than two admissions in ≥ 48 hours at term and not in established labour	
• Malpresentation after 37 completed weeks	• Any itchy rash		
• Placenta praevia	• Hb < 10 g/dL		
• Preterm labour before 37 completed weeks			

**Table 3:** Intrapartum transfer criteria

Maternal	Maternal	Fetal	Fetal
• Placental abruption	• Shoulder dystocia	• Abnormal fetal heart rate on auscultation - if prolonged deceleration ≥ 2 mins < 110 bpm is diagnosed, the woman was transferred to the CLU	• Meconium stained liquor
• Pyrexia > 38°C on two occasions at least 1 hour apart	• Request for epidural		• Malpresentation (with exception of mento-anterior)
• Lack of progress in the first stage of labour (absent or slower cervical dilatation than 0.5 cm/hr for primigravidae and 1 cm/hr for multigravidae	• Unbooked and presenting in early labour		• Intrapartum haemorrhage
• Delay in the second stage of labour (active pushing for more than 90 mins primigravidae or 40 mins for multigravidae)	• Retained placenta (> 1 hr)		• Cord presentation/prolapsed
	• PPH (> 1000 mls or if symptomatic)		• Fetal demise
	• 3^rd^/4^th ^degree perineal tears		• Absence of liquor

**Table 4:** Postnatal transfer criteria

Maternal	Maternal	Maternal
• Postpartum haemorrhage (> 500 mls)	• Signs of deep vein thromboembolism: leg pain or discomfort, (especially in the left leg), swelling, tenderness, increased temperature and oedema, and lower abdominal pain	• Signs of pulmonary thromboembolism: dyspnoea, collapse, chest pain, haemoptysis, faintness, and signs and symptoms associated with DVT
• Pyrexia > 38^C ^on two occasions at least 1 hour apart		• Any other condition that causes concern
• Concerns for psycho-logical wellbeing		

Data were collected manually from women's and neonates' charts by research assistants and double-entered into a computerised database, checked and cleaned. Verified data were transferred into SPSS (version 16.0) for analysis.

### Statistical analysis

MidU contained several primary outcomes, reflecting the diversity of opinion about which outcomes are most important in maternity care. The sample size required was 1,539, taking account of the two to one randomisation ratio and based on two-tailed tests. This assumed a criterion for significance (alpha) of 0.05, and sufficient power (at ≥ 0.80) to detect differences of at least 6% between consultant-led care and midwife-led care in MLUs in rates of induction of labour (23% to 17%), episiotomy (31% to 24%) and augmentation of labour (24.4% to 17.9%). Effect sizes for primary outcome measures were informed by the literature and agreed by study site clinicians. This sample size would also, with the same or greater levels of significance and power, detect differences in proportions between CLU and MLU in Apgar score at five minutes of 8-10: (97.2% to 93.2%); initiation of breastfeeding: (40% to 50%); CS: (11.2% to 6.2%); continuous electronic fetal monitoring (EFM): (23% to 16%); instrumental birth: (10.4% to 5.4%); postpartum haemorrhage (> 500 mls): (8% to 4%); and mean umbilical cord pH: mean difference of 0.02 with a common within-group standard deviation of 0.096. Secondary outcomes were also identified and listed in the study protocol.

Data analysis was by 'intention-to-treat'. Study results are reported as a summary of the outcome in each group (with denominators being the number of women randomised), or the mean and standard deviation of measurements. Summary statistics using risk ratios (relative risks (RR)) for dichotomous outcomes, and mean difference for continuous outcomes are reported, with 95% confidence intervals (CI) [18].

The protocol for the MidU study was registered with the International Standard Randomised Controlled Trial Number Register (ISRCTN14973283, http://www.controlled-trials.com/ISRCTN14973283) and no important changes were made to the methods after the trial started. Results are reported in accordance with the latest CONSORT statement [19-21].

## Results

**Figure 1:**
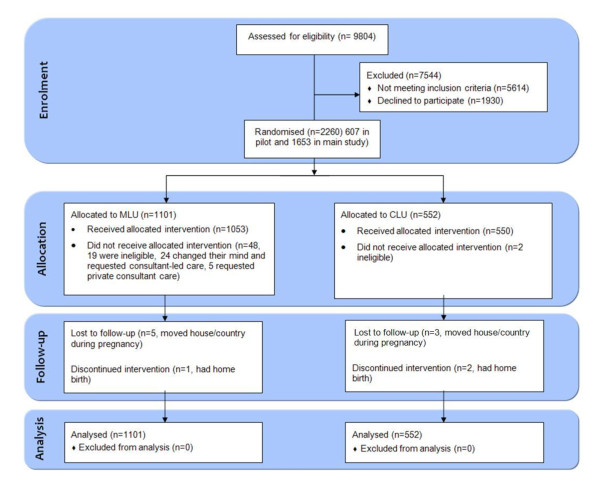
**Enrolment flowchart**.

Of the 9,804 women informed about the study, 4,190 (43%) were eligible to participate. Fifty-four percent (n = 2260) consented to join, 607 in the pilot and 1,653 in the main study. In the main study, reported here, 1,101 were randomised to MLU and 552 to CLU. Of the 1,653 women, 1,206 attended OLOL (73%) and 447 attended CGH (27%). Baseline characteristics were similar (Table 5). Data for five MLU women (0.5%) and three CLU women (0.5%) were incomplete because they moved home during pregnancy and could not be traced. Nineteen ineligible women were randomised to MLU (1.7%) and therefore transferred to CLU, but their data were analysed in the MLU group, in accordance with the intention-to-treat principle. Two ineligible women were randomised to CLU (0.4%). Twenty-four women (2.2%) randomised to MLU changed their minds following randomisation and requested CLU care. Five women randomised to MLU (0.5%) requested private consultant care. One woman randomised to MLU (0.1%) and two women randomised to CLU (0.4%) opted for home-birth (Figure 1). In all, 492 (44.7%) women transferred permanently to CLU in the antenatal period, 144 (13.1%) during labour and 5 (0.5%) in the postnatal period, based on *a priori *criteria (Tables 2, 3 and 4). The most common reason for transfer antenatally was for induction of labour (n = 202, 41%), with fetal assessment (n = 38, 8%) as the next most common reason. In labour, the most common reasons for transfer were slow progress (n = 61, 41%) and meconium stained liquor (n = 26, 18%).

**Table 5:** Baseline characteristics

	MLUs	CLUs
**Total**	1101	552

**Mean age years (SD)**	29 (4.9)	28.7 (5.0)

**Parity 0 (%)**	565 (51.3)	276 (50)

**Parity > 0 (%)**	536 (48.7)	276 (50)

**Single (%)**	415 (37.7)	229 (41.5)

**Married, not separated (%)**	664 (60.3)	312 (56.5)

**Mean weight Kgs (SD)**	65.9 (8.9)	66.1 (8.93)

**Mean height metres (SD)**	1.66 (0.07)	1.66 (0.08)

SD = Standard deviation

In accordance with the intention-to-treat principle, data are analysed in the group to which the women were allocated by randomisation. Seven primary outcomes showed no statistically significant difference between MLU and CLU: caesarean birth (163 [14.8%] *vs *84 [15.2%]; relative risk (RR) 0.97, 95% CI 0.76, 1.24), induction of labour (248 [22.5%] *vs *138 [25.0%]; RR 0.90, 95% CI 0.75, 1.08), episiotomy (126 [11.4%] *vs *68 [12.3%]; RR 0.93, 95% CI 0.70, 1.23), instrumental birth (139 [12.6%] *vs *79 [14.3%]; RR 0.88, 95% CI 0.68, 1.14), Apgar scores less than 8 (10 [0.9%] *vs *9 [1.6%]; RR 0.56, 95% CI 0.23, 1.36), postpartum haemorrhage (PPH) (144 [13.1%] *vs *75 [13.6%]; RR 0.96, 95% CI 0.74, 1.25) and initiation of breastfeeding (616 [55.9%] *vs *317 [57.4%]; RR 0.97, 95% CI 0.89, 1.06) (Table 6).

**Table 6:** Primary outcomes

Outcome	MLU (1101) Event [%]	CLU (552) Event [%]	Summary statistic 95% CI
Caesarean section	163 [14.8%]	84 [15.2%]	RR 0.97, 95% CI 0.76, 1.24

Induction of labour	248 [22.5%]	138 [25.0%]	RR 0.90, 95% CI 0.75, 1.08

Episiotomy	126 [11.4%]	68 [12.3%]	RR 0.93, 95% CI 0.70, 1.23

Instrumental birth	139 [12.6%]	79 [14.3%]	RR 0.88, 95% CI 0.68, 1.14

Apgar scores less than 8	10 [0.9%]	9 [1.6%]	RR 0.56, 95% CI 0.23, 1.36)

Postpartum haemorrhage (PPH)	144 [13.1%]	75 [13.6%]	RR 0.96, 95% CI 0.74, 1.25

Initiation of breastfeeding	616 [55.9%]	317 [57.4%]	RR 0.97, 95% CI 0.89, 1.06

Continuous EFM	397 [36.1%]	313 [56.7%]	RR 0.64, 95% CI 0.57, 0.71

Labour augmentation	436 [39.6%]	314 [56.9%]	RR 0.50, 95% CI 0.40, 0.61

MLU women were significantly less likely to receive continuous EFM (available only in CLU) (397 [36.1%] *vs *313 [56.7%]; RR 0.64, 95% CI 0.57, 0.71), or have labour augmented by amniotomy or with oxytocin (436 [39.6%] *vs *314 [56.9%]; RR 0.50, 95% CI 0.40, 0.61) (Table 6). Cord blood pH measurement could not be undertaken without cord-clamping, which was not part of the protocol of care in the MLUs (because cord-clamping would have excluded expectant third stage management).

Of the secondary maternal outcomes, 6 showed no statistically significant difference between MLU and CLU: at least one antenatal admission (487 [44.2%] *vs *229 [41.5%]; RR 1.07, 95% CI 0.95, 1.20); experienced any pregnancy complication (248 [22.5%] *vs *110 [19.9%]; RR 1.13, 95% CI 0.93, 1.38); fetal loss before 24 weeks (17 [1.54%] *vs *5 [0.91%]; RR 1.70, 95% CI 0.63, 4.60); spontaneous vaginal birth (761 [69.1%] *vs *372 [69%]; RR 1.03, 95% CI 0.96, 1.10); intact perineum (421 [38.2%] *vs *225 [40.8%]; RR 0.96, 95% CI 0.85, 1.09); estimated mean blood loss (323 mls (SD 317) *vs *324 mls (SD 401); MD 6.17, 95% CI-32.12, 44.46) (Table 7).

**Table 7:** Secondary outcomes

Outcome	MLU (1101) Event [%]	CLU (552) Event [%]	Summary statistic 95% CI
Ultrasound examinations	1.98 (1.4)	2.49 (1.8)	MD -0.51, 95% CI -0.68, -0.34

Antenatal cardiotocographs	2.38 (3.6)	3.39 (3.8)	MD -1.01 95% CI -1.39, -0.63)

Antenatal admission	487 [44.2%],	229 [41.5%]	RR 1.07, 95% CI0.95, 1.20

Pregnancy complications	248 [22.5%]	110 [19.9%]	RR 1.13, 95% CI 0.93, 1.38

Fetal loss prior to 24 weeks	17 [1.5%]	5 [0.9%]	RR 1.70, 95% CI 0.63, 4.60

Epidurals	202 [18.3%]	134 [24.3%]	RR 0.76, 95% CI 0.62, 0.92

Included transcutaneous electrical nerve stimulation (TENS)	64 [16.0%]	170 [12.0%]	RR 1.33, 95% CI 1.02, 1.74

Hydrotherapy	257 [23.3%]	18 [3.3%]	RR 7.16, 95% CI 4.49, 11.42

One or two caregivers	297 [27.0%]	94 [17.0%]	RR 1.58, 95% CI 1.29, 1.95

Length of labour (hrs)	4.6 (3.3)	4.0 (2.4)	MD 0.53, 95% CI 0.25, 0.81)

Spontaneous pushing	726 [65.9%]	308 [55.8%]	RR 1.18, 95% CI 1.08, 1.29

Upright positions for birthing	300 [27.2%]	55 [10.0%]	RR 2.73, 95% CI 2.09, 3.58

Physiological management of third stage of labour	137 [12.4%]	1 [0.2%]	RR 68.69, 95% CI 9.63, 489.80

Spontaneous vaginal birth	761 [69.1%]	372 [69.0%]	RR 1.03, 95% CI 0.96, 1.10

Intact perineum	421 [38.2%]	225 [40.8%]	RR 0.96, 95% CI 0.85, 1.09

Estimated blood loss mls	323 (317)	324 (401)	MD 6.17, 95% CI -32.12, 44.46

Postnatal stay of 1 day or less	184 [16.7%]	57 [10.3%]	RR 1.62, 95% CI 1.22, 2.14

Paediatric care required	292 [26.5%]	150 [27.2%]	RR 0.98, 95% CI 0.82, 1.15

Facial oxygen	130 [11.8%]	63 [11.4%]	RR 1.03, 95% CI 0.78, 1.37

Bag-and-mask resuscitation	23 [2.1%]	12 [2.2%]	RR 0.96, 95% CI 0.48, 1.92

Admission to special care baby unit	128 [11.6%]	60 [10.9%]	RR 1.07, 95% CI 0.80, 1.43

Women randomised to MLU had significantly fewer mean ultrasound examinations (1.98 (SD 1.37) *vs *2.49 (SD 1.75), mean difference (MD) -0.51, 95% CI -0.68, -0.34), and antenatal cardiotocographs (2.38 (SD 3.6) *vs *3.39 (SD 3.77), MD -1.01 95% CI -1.39, -0.63). Significantly fewer MLU women chose to have epidurals (for which they had to transfer to CLU) (202 [18.3%] *vs *134 [24.3%]; RR 0.76, 95% CI 0.62, 0.92). Alternative methods of pain relief included transcutaneous electrical nerve stimulation (TENS) (64 [16%] *vs *170 [12%]; RR 1.33, 95% CI 1.02, 1.74 and hydrotherapy (birthing pool in MLU, bath in CLU) (257 [23.3%] *vs *CLU 18 [3.3%]; RR 7.16, 95% CI 4.49, 11.42). Twenty-seven percent of MLU women (n = 297) had only one or two caregivers in labour, compared with 17% (n = 94) in CLU (RR 1.58, 95% CI 1.29, 1.95). MLU women had a longer mean length of labour (4.6 hours (SD 3.27) *vs *4.0.hours (SD 2.41); mean difference 0.53, 95% CI 0.25, 0.81) (Table 7). MLU women more frequently used spontaneous pushing (726 [65.9%] *vs *308 [55.8%]; RR 1.18, 95% CI 1.08, 1.29), upright positions for birthing (300 [27.2%] *vs *55 [10.0%]; RR 2.73, 95% CI 2.09, 3.58), physiological management of third stage of labour (137 [12.4%] *vs *1 [0.2%]; RR 68.69, 95% CI 9.63, 489.80). More MLU women stayed only one postnatal day or less (184 [16.7%] *vs *57 [10.3%]; RR 1.62, 95% CI 1.22, 2.14) (Table 7).

Neonatal outcomes showed no statistically significant difference between MLU and CLU in paediatric care required (292 [26.5%] *vs *150 [27.2%]; RR 0.98, 95% CI 0.82, 1.15); facial oxygen (130 [11.8%] *vs *63 [11.4%]; RR 1.03, 95% CI 0.78, 1.37); bag-and-mask resuscitation (23 [2.1%] *vs *12 [2.2%]; RR 0.96, 95% CI 0.48, 1.92); admission to special care baby unit (SCBU) (128 [11.6%] *vs *60 [10.9%]; RR 1.07, 95% CI 0.80, 1.43) (Table 7). There were two early neonatal deaths in MLU (0.18%), two (0.36%) in CLU, and one (0.1%) fetal loss at > 24 weeks in MLU. Perinatal mortality rates in MLU and CLU were 2.76 and 3.66 per 1,000 live and still-births. There were no maternal deaths. Other secondary and sub-group analyses are in the full study report [22]. The satisfaction survey and economic analysis will be reported separately.

## Discussion

The trial's main limitation is the lack of blinding of participants and carers as all women attending MLU were known to be in the study intervention group. Those allocated to CLU care were not masked either, as the blinding of participants allocated to control groups in such situations has been criticised [17]. Unavoidable potential bias thus exists for both randomised groups. Assessors for certain outcomes, such as laboratory tests, were blinded to study group. The outcome 'blood loss' was estimated, as per hospital protocols, and amounts are thus imprecise in both groups.

The focus of this study was on the relative effects of midwife-led care provided in the setting of an alongside MLU. As such, this study combines elements of midwife-led care including continuity of care in pregnancy and birth with settings for birth i.e. the MLU. We acknowledge that not all midwife-led models of care will take place in an alongside MLU nor, indeed, in a home-like environment [7]. Further, not all alternative settings for birth will provide midwife-led care [23]. Differentiating the effects of midwife-led care from the setting of that care is not possible within this study, a limitation that is not unique to our study. The potential confounding effect of practice settings such as MLU on the outcomes of midwife-led care is complex as are the interrelationships between philosophy and continuity of care [7].

In this study, the percentage of women transferring from MLU to CLU care in the antenatal period, in particular, is higher (at 45%) than quoted rates of 24% in some UK centres [10]. The permanent transfer rates of 13% intrapartum and 0.5% postnatally are approximately the same as the 12-15% and up to 8% reported in the UK [10]. Some of the reasons for permanent transfer such as induction of labour and premature labour should not automatically preclude women from being transferred back to MLU care in the postnatal period, if appropriate. Quality reviews and audits of reasons for transfer would assist in reducing these high rates to more normal levels.

The strength of this trial lies in its size, and in its serendipitous conduct prior to the introduction of MLUs, in Ireland, due to the enlightened vision of the (then) North-Eastern Health Board in planning service innovation formally within the framework of a clinical trial. The MidU study is likely to have good external validity, as the setting of the trial is similar to many birthing units in the UK and other countries. Identifying eligible women was done using clinical criteria of 'low risk' similar to, or sometimes more stringent than, those used in many other centres, and 43% of women met those criteria. As 50% of these women also agreed to join the trial, there is a large enough proportion to warrant introducing a new scheme. Furthermore, the MLU intervention described in the trial protocol and implemented in the trial itself for women allocated to the MLU arm can be regarded as standard practice for MLU care in the two study sites, since the relevant procedures were established in the context of the MidU trial. Similarly, the control intervention in MidU reflects standard practice for women receiving CLU care in these two sites.

We calculated the sample size for the MidU trial using estimates for induction of labour, episiotomy and augmentation of labour. We also calculated the effect sizes that this would allow us to detect for other important outcomes and included this information in the protocol for the trial and within this report. We thus had several "primary outcomes" because complex interventions such as models of maternity care involve a variety of people including pregnant women, practitioners, policy makers and the public, all of whom are likely to be interested in the results of a randomised trial. These people can have different priorities when assessing the evidence from a trial such as MidU, and it is unclear whose priorities should be given prominence by choosing a single primary outcome. This diversity of opinion was confirmed in many discussions before, during and after the trial and, so, by explicitly selecting several primary outcomes, we committed ourselves to making the findings for each of these available in the report of the study, including all adverse outcomes, as we have done here. In this way, we leave it to readers to use their own priorities to decide whether any single outcome, or combination of outcomes, is the most important for their interpretation of the findings of MidU.

The results reported here show that midwife-led care, as practised in this study in an 'alongside' MLU, is as safe as consultant-led care and is associated with less intervention. Women cared for in the MLUs were significantly less likely to receive continuous EFM, or have their labour augmented, with no statistically significant difference in adverse neonatal or maternal outcomes such as low Apgar scores, resuscitation, admission to SCBU, CS, instrumental birth or PPH. Other intervention rates, such as episiotomy and induction of labour, were similar in both groups.

The lower rate of EFM in the MLUs should be seen in the context of knowledge that the predictive ability of abnormal fetal heart rate patterns to identify fetal metabolic acidosis and hypoxic-ischemic encephalopathy is low [24]. The Cochrane Review of continuous cardiotocography (CTG) during labour found that, in 12 trials with more than 37,000 women, continuous EFM was associated with a significant increase in CS rates and instrumental birth with no difference shown in cerebral palsy or neonatal mortality. EFM was, however, associated with a reduction in neonatal seizures [25]. Despite the significantly lower use of EFM in women randomised to MLU, there was no difference in the CS rates between the groups. Although counter-intuitive, this finding is consistent with the Cochrane Review on midwife-led care [7]. The lower rate of instrumental vaginal births is consistent with the Cochrane Reviews on both continuous CTG [26] and midwife-led care [7].

Augmentation of labour, through 'active management,' was introduced in the 1970s in Ireland to prevent prolonged primigravid labours and save 60% on birth costs [26]. A review comparing routine care with early amniotomy and oxytocin for delay in first stage spontaneous labour found that early intervention was associated with a modest reduction in CS rates but there was insufficient evidence on maternal or neonatal outcomes [27]. The higher augmentation rates for women in CLU in this study are not associated with a reduction in complications in the fetus or neonate, or with a decrease in operative or instrumental birth rates, and are therefore unnecessary in low-risk women.

Recent editorial commentary in the Lancet recommended that trialists should set their findings in the context of an up-to-date systematic review, in order to acknowledge the place of their results in the world literature [28]. In line with this recommendation, a comparison of MidU primary and some secondary outcome results with the Cochrane Review of midwife-led versus consultant-led care was conducted, [7] and shows some differences but many similarities. Only those results where the addition of the MidU data did not lead to substantial increases in the I^2 ^statistic for heterogeneity (greater than I^2 ^= 75%) have been presented.

The addition of the MidU findings strengthens the findings of the review, increasing the statistical power of the meta-analyses and changing some results from statistically significant to non-significant for the secondary outcomes of 'antenatal hospitalisation' (RR 0.90 95% CI 0.81, 0.99 I^2 ^= 32% without MidU, to RR 0.96 95% CI 0.89, 1.03 I^2 ^= 49% with MidU); and 'fetal/neonatal loss before 24 weeks' (RR 0.79 95% CI 0.65, 0.97 I^2 ^= 0% without MidU, to RR 0.82, 95% CI 0.67, 1.00 I^2 ^= 0% with MidU). The addition of MidU also changes the results of the meta-analyses from non-significant to significant in favour of MLU for the outcome 'amniotomy' (RR 0.88 95% CI 0.75, 1.04 I^2 ^= 41% without MidU; to RR 0.80 95% CI 0.66, 0.97 I^2 ^= 74% with MidU), and in favour of CLU for the outcome 'shorter mean length of labour' (mean difference 0.27 95% CI -0.18, 0.72 I^2 ^= 0% without MidU to mean difference 0.46 95% CI 0.22, 0.70 I^2 ^= 0% with MidU). As in MidU, four studies included in the Cochrane Review [7] found significant reduced rates of augmentation of labour in MLUs, but the remaining six showed no difference. Overall, meta-analyses do not show a significant difference in this outcome, with or without the addition of MidU data [6]. In the MidU study, induction of labour, PPH and low Apgar scores were not different between the two groups, in accord with the Cochrane Review findings. The MidU study, in common with eight others in the review, showed a reduction in instrumental birth in MLUs, which was not statistically significant. The increased power available in the Cochrane meta-analysis, before and after addition of the MidU data, continues to show a significant decrease in instrumental birth rates in favour of MLUs [7]. Similarly, the MidU study, in common with five others in the Cochrane Review, showed a non-significant reduction in episiotomies for MLU women. One other study in the review showed no difference, but five showed a statistically significant difference in favour of MLU care. The increased power available in the Cochrane meta-analysis, before and after addition of the MidU data, shows a significant difference in episiotomy rate in favour of MLUs [7]. Consistent with MidU, the Cochrane Review finds no significant difference in 'fetal loss/neonatal death equal to/after 24 weeks' before and after the addition of MidU data.

In summary, MidU adds to the totality of evidence available by substantiating and adding to the results of previous research. The similarity between MidU and other international studies in the Cochrane Review shows that our findings have good generalisability. Further research is necessary into ways of decreasing non-essential interventions for healthy women in normal labour, to increase the normal birth rate for healthy women at low risk to complications.

## Conclusions

This study supports the recommendation of the Cochrane Review [7] that midwife-led care should be offered to most women. The implications of this for maternity care are profound, particularly in Ireland where at least 40% of women are eligible, and suitable, for midwife-led care (using strict criteria), which has been shown to be as safe as consultant-led care but associated with less intervention when provided in an 'alongside' MLU. Therefore, it is reasonable to suggest that midwife-led care should be the norm for low-risk women. Consideration should be given to the establishment of MLUs where they are not the norm, using the model of midwife-led care practised in this study, with similar resources.

## List of abbreviations used

MLU: Midwife-led unit; CLU: Consultant-led unit; RR: Relative risk; CI: Confidence interval; GP: General practitioner; AML: Active management of labour; ARM: Artificial rupture of membranes; CS: Caesarean section; NEHB: North-Eastern Health Board; HSE-DNE: Health Service Executive, Dublin North-East; OLOL: Our Lady of Lourdes Hospital; CGH: Cavan General Hospital; DSMB: Data and Safety Monitoring Board; SPSS: Statistical package for the Social Sciences; EFM: Electronic fetal monitoring; PPH: Postpartum haemorrhage; TENS: Transcutaneous electrical nerve stimulation; PUPP: Pruritic urticarial papules and plaques

## Competing interests

All authors declare that (1) DD and CB had support from the HSE-DNE for the submitted work [CB was awarded funding to her institution to conduct the MidU study. She personally received travel expenses to travel to a research conference to present the literature review and methodology. DD was the research assistant on the MidU study and received a PhD student stipend and travel expenses from the funding awarded]; (2) CB was awarded other grants by the HSE-DNE, during the time of the MidU study, to conduct other studies. For the period 2002-2010, she was a member of the Maternity Services Taskforce in the HSE-DNE and received travel expenses to attend meetings every 6-8 weeks; (3) RM, at the time of the MidU study and at present, is an employee of the HSE-DNE; all other authors, their spouses, partners, or children have no financial relationships that may be relevant to the submitted work; and (4) all authors have no non-financial interests that may be relevant to the submitted work.

## Authors' contributions

CB, DD and MC contributed to the conception of the trial and data analysis. CB, DD, MC, CMcC, PH, MR, RM, SH, AF, SG, MD participated in the study design and implementation. CB, DD and MC drafted the manuscript with contributions from CMcC, PH, MR, RM, SH, AF, SG and MD. All authors read and approved the final manuscript.

## Pre-publication history

The pre-publication history for this paper can be accessed here:

http://www.biomedcentral.com/1471-2393/11/85/prepub
